# Parasitoid Wasps Can Manipulate Host Trehalase to the Benefit of Their Offspring

**DOI:** 10.3390/insects13090833

**Published:** 2022-09-13

**Authors:** Yan Song, Fengming Gu, Weihong Zhou, Ping Li, Fuan Wu, Sheng Sheng

**Affiliations:** 1 School of Biotechnology, Jiangsu University of Science and Technology, Zhenjiang 212100, China; 2 The Key Laboratory of Silkworm and Mulberry Genetic Improvement, Ministry of Agriculture and Rural Affairs, Sericultural Research Institute, Chinese Academy of Agricultural Science, Zhenjiang 212100, China

**Keywords:** *Spodoptera litura*, *Meterous pulchricornis*, trehalase, parasitoid offspring fitness

## Abstract

**Simple Summary:**

Trehalase plays a vital role in carbohydrate metabolism in insects. However, less attention has been paid to its role in the interaction between parasitoid wasps and their hosts. Here, we found that two trehalase genes, *SlTre1* and *SlTre2*, were highly expressed in the third instar larvae of *Spodoptera litura* after they were parasitized by *Meterous pulchricornis*. Furthermore, we silenced *SlTre1* and *SlTre2* in parasitized *S. litura* larvae, and after that, the activity of trehalase and the content of glucose of the host larvae were decreased significantly. In addition, after knocking down *SlTre1* or *SlTre2 i*n parasitized *S. litura* larvae, the fitness of parasitoid offspring was negatively affected. The results obtained here suggested that parasitoid wasps can induce the upregulation of trehalase in their host larvae and support the development of their offsprings. The present study provides a theoretical base for functional research on *trehalase* genes in the coevolution between parasitoid wasps and their hosts.

**Abstract:**

Trehalase is an essential hydrolase of trehalose in insects. However, whether and how trehalase performs in the association of parasitoid wasps and their hosts still remains unknown. Here, the exact function of trehalase of the general cutworm *Spodoptera litura* after it was parasitized by its predominant endoparasitoid *Meterous pulchricornis* was elucidated. Two trehalase genes (*SlTre1*, *SlTre2*) were identified, and they were highly expressed five days after parasitization by *M. pulchricornis*. Then, we successfully silenced *SlTre1* and *SlTre2* in parasitized third instar *S. litura* larvae. The content of glucose, which is the hydrolysate of trehalose, was significantly decreased after silencing *SlTres* in parasitized *S. litura* larvae, and the activities of trehalase were also notably reduced. In addition, the cocoon weight, the emergence rate, proportion of normal adults, and the body size of parasitoid offsprings were significantly decreased in *SlTre1*- or *SlTre2*-silenced groups compared to the controls. These results implied that parasitization by parasitoids regulated the trehalase of host larvae to create a suitable nutritional environment for the parasitoid offspring. The present study broadens the knowledge of trehalase in the interaction between parasitoids and their hosts and is of benefit to biological control of *S. litura* acting by parasitoid wasps.

## 1. Introduction

Trehalose is a non-reducing disaccharide and is ubiquitous in organisms. In insects, trehalose forms the major hemolymph sugar and is synthesized in the fat body by trehalose 6-phosphate synthase (TPS; EC 2.4.1.15) and trehalose 6-phosphate phosphatase (TPP; EC 3.1.3.12) [1,2]. Trehalose is usually hydrolyzed by trehalase (Tre; EC 3.2.1.28) to refuel the energy requirement for various physiological activities or behaviors, and it is the only reported hydrolase of trehalose in all organisms at present [2]. There are two distinct forms of trehalase existing in insects, Tre1 as the soluble form and Tre2 as the membrane-bound form [2].

Insect trehalose is converted into glucose by trehalase, and this stringent control directly releases energy to maintain flight, growth, metamorphosis, and reproduction in insects [2]. For instance, the inhibitor trehazolin can inhibit Tre-2 in the flight muscles of *Locusta migratoria* and lead to serious hypoglycemia. This shortage of sugar supply can be compensated by feeding the locust glucose, suggesting the key role of trehalase in meeting the energy requirements during insect flight [3]. Silencing trehalase genes, *LsTre-1* and *LsTre-2*, not only causes lethal effects but also ceases normal growth and development in the small brown plant hopper *Laodelphax striatellus* [4]. Chen et al. [5] found that knocking down *Tre-1* and *Tre-2* in the larvae and pupae of *Spodoptera exigua* produced more severe abnormal phenotypes, such as abnormal abdomen, misshapen-wings, and half-eclosion. A close linkage between Tre-2 and oogenesis in the silkworm *Bombyx mori* was demonstrated, and it was hypothesized that membrane-bound trehalase is essential in sugar accumulation in the embryonic stages [6].

Recent studies also revealed that trehalase is responsible for abiotic stressor tolerance in insects. As a non-reducing sugar, trehalase stabilizes cellular membranes and protects proteins by replacing water molecules and facilitating cytosolar vitrification [1]. Plenty of studies have demonstrated that trehalase plays essential roles in desiccation tolerance in insect species such as *Polypedilum vanderplanki*, *Belgica Antarctica*, and *Drosophila melanogaster* [7,8,9]. Another conspicuous role of trehalase is the contribution to insecticide tolerance in insects. It is accepted that trehalase could reduce the toxicity of insecticides by regulating the energy supply reaction and protecting proteins and cytoplasms [10,11,12]. For example, in the deltamethrin-resistant strain of *Culex pipiens*, the expression of the trehalase gene *Tre1* was significantly upregulated, and after silencing *Tre1*, the mortality of the deltamethrin-resistant mosquitos was increased, suggesting the crucial role of trehalase in deltamethrin resistance in *C. pipiens* [13].

Apart from the role of abiotic stressor tolerance of trehalase, its roles in response to biotic stress still receive less attention. Parasitoid wasps are natural enemies of insect pests. They lay their eggs into or onto their host insects, and after hatching, the parasitoid larvae feed on the hosts and eventually kill them [14]. In order to create a safe and suitable environment for their offspring, maternal wasps inject various so-called parasitization factors, such as venom and polydnavirus into the cavities of hosts to overcome the hosts’ immune response [15,16,17]. In addition, host nutrition metabolism manipulation is deemed to be another mission for parasitoid wasps [15]. Specifically, the endoparasitoids (lay their eggs into the host body and the infants develop within the host cavity) should more precisely manipulate the nutrition dynamics of hosts to balance the development between hosts and themselves [14]. Previous studies demonstrated that parasitization by endoparasitoids could slow down the development of hosts by suppressing the activity of enzymes in sugar or lipid metabolism [14]; however, the detailed molecular mechanism is not fully understood. The impact of parasitization on host trehalase also receives less attention.

The general cutworm *Spodoptera litura* (Lepidoptera: Noctuidae) is one of the most destructive pests of soybean, cotton, and vegetable crops [18]. *Meterous pulchricornis* (Hymenoptera: Braconidae) is a predominant endoparasitoid of *S. litura* larvae [19]. Previous studies have revealed the characterizations of two trehalase genes in *S. litura* [19] and their roles in response to the trehalase inhibitor Validamycin [20]; however, the effect of parasitization by parasitic wasps on trehalase of *S. litura* has not been studied until now. In the present study, we identified two trehalase genes from a previously constructed *S. litura* transcriptome database and explored their functions in response to parasitization by *M. pulchricornis*. The results obtained here shed light on the understanding of the role of trehalase in the interaction between parasitoid wasps and their hosts and provide novel targets for the integrated management of *S. litura*.

## 2. Materials and Methods

### 2.1. Insect Rearing and Parasitization

*S. litura* larvae were obtained from mulberry fields at the campus of Jiangsu University of Science and Technology, Zhenjiang city, Jiangsu province, China, reared in the insectary [26 ± 2 °C, 60–80% relative humidity, and photoperiod of 14:10 (L:D) h], and fed with artificial diets [21]. The endoparasitoid wasp *M. pulchricornis* was originated from the parasitized *S. litura* larvae in the mulberry field and was maintained using third instar *S. litura* larvae as host insects [21]. The adult wasps were reared in glass tubes (2.2 cm diameter × 8 cm height), and 100 μL 10% (*w*/*w*) honey solution was supplied via cotton lines every day.

For the parasitization assay, 15–20 third instar *S. litura* larvae were exposed to one female *M. pulchricornis* in a transparent plastic box (6 cm diameter × 3 cm height) with a circular mulberry leaf at the bottom of the box. After release of the female wasp into the transparent plastic box, the behavior of parasitization was observed and recorded by the observers directly. The female wasp exhibits a featured parasitization behavior in which it stung the ovipositor into the host body for several seconds [21], and once the behavior of parasitization was observed, we collected the *S. litura* larvae and reared them individually in petri dishes (6 cm diameter) and marked them as parasitized hosts.

### 2.2. Identification of SlTre Genes and Bioinformatics Analysis

The sequences of SlTre were identified from the previously constructed *S. litura* transcriptome database (BioProject Acc. in NCBI: PRJNA810583) (accessed on 15 January 2022). The Open Reading Frame (ORF) Finder (https://www.ncbi.nlm.nih.gov/orffinder/) (accessed on 15 January 2022) was used to predict the ORFs of putative *SlTre1* and *SlTre2* genes. ExPASy (https://web.expasy.org/compute_pi/) (accessed on 15 January 2022) was used to predict the theoretical isoelectric point (pI) and molecular weight (MW) of SlTre1 and SlTre2. DNAMAN 8.0 (Lynnon Corporation, Quebec City, QC, Canada) was used to perform multiple alignment and homology analysis of various protein sequences. Phylogenetic analysis was conducted using Molecular Evolutionary Genetic Analysis 6.0 (MEGA 6.0) (Mega Limited, Auckland, New Zealand) with the neighbor-joining method and 1000 bootstrap replications. SlitTre homologous protein sequences from *Spodoptera litura* (Sl), *Tribolium castaneum* (Tc), *Bombyx mori* (Bm), *Aphis glycines* (Ag), *Spodoptera exigua* (Se), *Helicoverpa armigera* (Ha), *Aedes aegypti* (Aa), *Apis mellifera* (Am), *Drosophila melanogaster* (Dm), *Locusta migratoria* manilensis (Lm), *Nilaparvata lugens* (Nl), *Omphisa fuscidentalis* (Of), *Cnaphalocrocis medinalis* (Cm), *Glyphodes pyloalis* (Gp), *Spodoptera frugiperda* (Sf), and *Anopheles gambiae* (Ag) were downloaded from GenBank (http://www.ncbi.nlm.nih.gov/) (accessed on 15 January 2022). GenBank accession numbers of sequences used were listed in Table S1. The interactive tree of life (iTOL) (https://itol.embl.de/) (accessed on 15 January 2022) was used to generate and annotate the circular phylogenetic tree.

### 2.3. Sample Collection, RNA Isolation and qRT-PCR Analysis

*S. litura* larvae samples were collected at 0 (at parasitism), 1, 3, and 5 days after parasitization by *M. pulchricornis*. Healthy third instar *S. litura* larvae were collected at the same time points and used as controls. Total RNA was extracted from the whole body of third instar *S. litura* larvae using TRIzol reagent kit (Invitrogen, Life Technologies, Grand Island, NY, USA) following the instructions of the manufacturer. RNA concentrations were determined using a 2100 Bioanalyzer (Agilent Technologies, California, CA, USA) to evaluate absorbance at 260 nm, and the purity of RNA was determined by the OD 260/280 ratio. The integrity of RNA was identified using 1.5% agarose gel electrophoresis. According to the manufacturer’s instructions, 1 μg total RNA was used to synthesize the first-strand cDNA using the PrimeScript^®^ RT reagent Kit (Takara, Dalian, China), followed by reverse transcription. All primers were designed using the Primer-BLAST on-line programme (https://www.ncbi.nlm.nih.gov/tools/primer-blast/) (accessed on 15 January 2022) (Table S2). The qRT-PCR reaction was conducted by using QuantStudio™ 6 Flex (Thermo Fisher Scientific, Waltham, MA, USA), and the total volume was 20 μL, including 10 μL 2 × iQTM SYBR^®^ Green I buffer, 1 μL 10 μM of each of the forward and reverse primers, 2 μL cDNA template, and 6 μL ultrapure water. The qRT-PCR program was as follows: 95 °C for 5 min, 35 cycles of 95 °C for 15 s, and 60 °C for 30 s. The no-template controls (NTCs) of each primer were negative with non-detection of the Cq value. The *glyceraldehyde-3-phosphate dehydrogenase* (*GAPDH*) gene (GenBank accession: MZ393966.1) and *elongation factor-1 alpha* (*EF1*) gene (GeneBank accession: DQ192234.1) were used as reference genes to normalize the expression levels of mRNA. The qRT-PCR results were analyzed by using LightCycler^®^ 96 software (Roche, Switzerland). The relative expression levels were calculated by using the 2^−ΔΔCt^ method [22]. Each treatment was run in triplicate for technical repeats, and three biological replicates were performed simultaneously.

### 2.4. RNA Interference of SlTres

To investigate the function of *SlTres* in *S. litura* larvae subjected to parasitization by female *M. pulchricornis*, *SlTre1* and *SlTre2* were selected for RNA interference. The oligonucleotide sequences of *SlTre1* and *SlTre2* were designed using BLOCK-iTTM RNAi Designer (https://rnaidesigner.thermofisher.com/) (accessed on 15 January 2022) (Table S3). dsRNA of *SlTre1* and *SlTre2* were synthesized by using the Transcription T7 kit (Taktableara Biotechnology Co. Ltd., Dalian, China) in vitro based on the manufacturer’s protocol, and the dsRNA of the green fluorescence protein gene (GFP) was set as a negative control. The NanoDrop 2000 spectrophotometer (Thermo Fisher Scientific, Waltham, MA, USA) was used to ensure the concentration purity of synthesized dsRNA, and the quality of dsRNA was detected using 1.5% agarose gel electrophoresis (BIO-RAD, Hercules, California, USA). Subsequently, dsRNA was diluted to the working concentration of 1000 ng/μL and stored at −80 °C until use.

One microliter of *dsSlTre1* and *dsSlTre2* (1000 ng) was injected into the third abdominal segment of third instar *S. litura* larvae on the fifth day after parasitization by female *M. pulchricornis* by using a Nanoject II micro syringe (Drummond Scientific, Broomall, PA, USA), respectively. The parasitized *S. litura* larvae were collected 24 and 48 h after injection of *dsSlTre1*, *dsSlTre2*, and *dsGFP*. Total RNA was extracted, and the cDNA was synthesized by using the method mentioned above. qRT-PCR was conducted to validate the expression levels of *SlTre1* and *SlTre2* after silencing and the procedure was the same as mentioned above. Each group contained three biological replicates.

To further investigate the effect of *SlTres* on the development of the offspring of *M. pulchricornis* egressed from the *SlTres*-silenced parasitized *S. litura* larvae, the duration from egg to cocoon (e.g., from oviposition to cocoon formation), cocoon weight, pupation rate, emergence rate, proportion of abnormal adults, hind tibia length, and longevity of offspring wasps were recorded. The cocoons were weighted by using an electronic balance (Ohaus, model AR224CN, New York, NY, USA, to an accuracy of 0.01 mg). The hind tibia length was measured under a microscope (Nikon, SMZ800N, Tokyo, Japan, to an accuracy of 0.001 mm) as the body size correlate. Briefly, the hind legs were dissected from individual wasp under the microscope and put on a glass slide, and then they were photographed and the lengths were measured by the image analysis software of the microscope (version 5.01.00, NIS-Elements D, Nikon, Maru, Chiyoda Ward, Tokyo, Japan). The method of RNAi was the same as described above. Each treatment was tested in 30 individual parasitized *S. litura* larvae, and the dsGFP injection groups were taken as the control.

### 2.5. Determination of Trehalose and Glucose Content

Trehalose content was determined by the anthrone-sulfuric acid colorimetric method using the Trehalose Assay kit (Beijing Grace Biotechnology Co. Ltd., Beijing, China). In brief, the *S. litura* larvae were collected and weighed by using an electronic balance (Ohaus, model AR224CN, New York, NY, USA, to an accuracy of 0.01 mg); then, the extraction liquid was added to the tube. Subsequently, the solution was shaken at room temperature for 30 min, and centrifuged at 8000× *g* for 10 min at 25 °C. Then, 300 μL of supernatant and 600 μL of reaction reagent were mixed, and incubated in boiling water (95–100 °C) for 3 min. After cooling to room temperature, all of the mixed solution was used to test trehalose content by reading absorbance at 620 nm using a spectrophotometer (Thermo1500, Waltham, MA, USA). Each sample contained three biological replicates.

The content of glucose was determined using the Glucose Assay kit (Beijing Grace Biotechnology Co., Ltd., Beijing, China) with the glucose oxidase–peroxidase method. Firstly, 0.1 g of weighted insects was homogenized in 1 mL of distilled H_2_O on ice and centrifuged at 12,000× *g* for 10 min at 25 °C. Then, 10 μL of supernatant was thoroughly mixed with 190 μL of reaction reagent solution and incubated at 37 °C for 30 min. Meanwhile, ddH_2_O and 1 mg/mL glucose solution were used as the control and standard, respectively. The mixed solution was calculated by measuring the absorbance at 520 nm using a spectrophotometer (Thermo1500, Waltham, MA, USA). Each sample contained three biological replicates. All glucose and trehalose contents were measured in terms of content per capita (total content divided by body weight).

### 2.6. Trehalase Activity Assay

The trehalase activity assay was determined according to the protocol described by Yang et al. [23] with some modification. Briefly, the third instar parasitized *S. litura* larvae were collected 24 and 48 h after dsRNA injection and placed in 1.5 mL Eppendorf tubes, and 1 mL phosphate-buffered saline (PBS: 130 mM NaCl; 7 mM Na_2_HPO_4_·2H_2_O; 3 mM NaH_2_PO_4_·2H_2_O; pH 7.0) was added. Subsequently, the sample was homogenized on ice then centrifuged at 1000× *g* for 20 min at 4 °C. Then, approximately 200 μL supernatant and 600 μL reaction regent were mixed, boiled at 95–100 °C for 5 min, and centrifuged at 12,000× *g* for 10 min at 4 °C. Then, the supernatant was boiled at 95–100 °C for 5 min and centrifuged at 12,000× *g* for 10 min at 4 °C. After that, the trehalase activity was measured by reading the absorbance of the supernatant at 520 nm using a Spectrophotometer (Thermo1500, Waltham, MA, USA) and the Trehalase Assay kit (Beijing Grace Biotechnology Co., Ltd., Beijing, China). Each group contained three biological replicates.

### 2.7. Statistical Analysis

One-way analysis of variance (ANOVA) was used to compare the differences of relative expression levels, the content of glucose and trehalose, activity of trehalase, and the fitness of parasitoid offspring. Chi-square test was used to compare the differences of duration from egg to cocoon. All data were analyzed using R version 4.0.0 (R Development Core Team, Vienna, Austria).

## 3. Results

### 3.1. Identification and Characterizations of Trehalase Genes in S. litura

**Figure 1:**
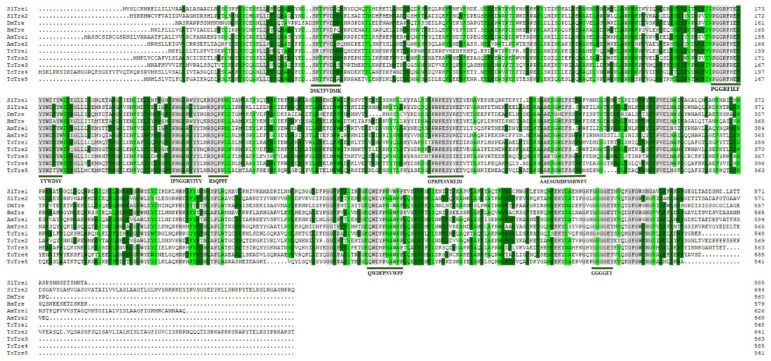
Amino acid sequence alignment of *S. litura* trehalase with its homologues in other species. Identical amino acids were highlighted in grey, and similar amino acids were highlighted in dark green and light green. Trehalase signature regions were indicated by the dark green solid lines. *Drosophila melanogaster* (Dm); *Bombyx mori* (Bm); *Apis mellifera* (Am); *Tribolium castaneum* (Tc); *Spodoptera litura* (Sl).

**Figure 2:**
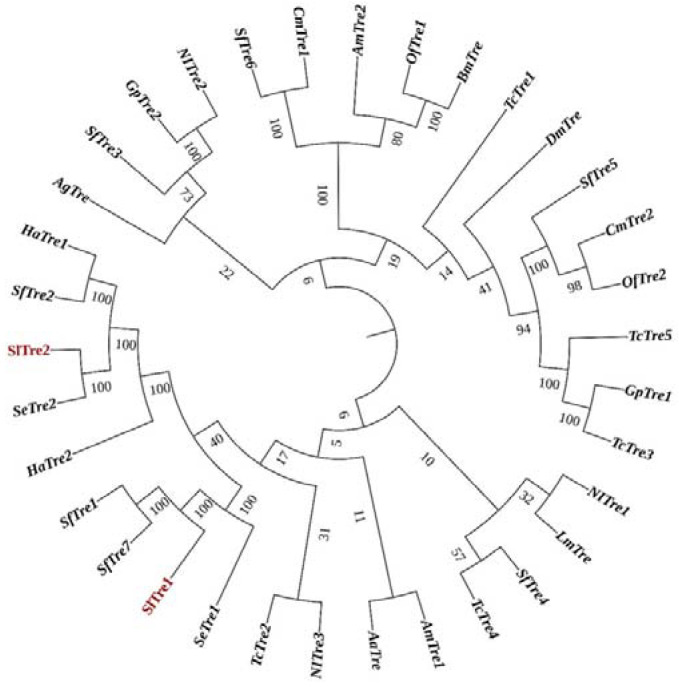
Phylogenetic analysis of trehalases in different species based on amino acid sequences. *Tribolium castaneum* (Tc), *Bombyx mori* (Bm), *Aphis glycines* (Ag), *Spodoptera exigua* (Se), *Helicoverpa armigera* (Ha), *Aedes aegypti* (Aa), *Apis mellifera* (Am), *Drosophila melanogaster* (Dm), *Locusta migratoria* manilensis (Lm), *Nilaparvata lugens* (Nl), *Omphisa fuscidentalis* (Of), *Cnaphalocrocis medinalis* (Cm), *Glyphodes pyloalis* (Gp), *Spodoptera frugiperda* (Sf), *Anopheles gambiae* (Ag), *Spodoptera litura* (Sl).

Two trehalase genes, *SlTre1* (LOC111362615) and *SlTre2* (LOC111362037), were identified from our previously constructed *S. litura* transcriptome database (BioProject Acc. in NCBI: PRJNA810583). The sequences of SlTre1 contained a complete ORF of 1758 bp, which encoded 585 amino acid residues, with a predicted mass of approximately 67.07 kDa and an isoelectric point of 4.84. The sequences of SlTre2 contained a complete ORF of 1938 bp, which encoded 645 amino acid residues, with a predicted mass of approximately 73.86 kDa and an isoelectric point of 5.97. Multiple alignment of amino acid sequences showed the presence of a glycine-rich region (GGGGEY) and two signature motifs or “tag structures” (PGGRFIEFYYWDSY and QWDFPNVWPP) among selected insect trehalase. Five other conserved sequences, DSKTFVDMK, IPNGGRV/IYY, RSQPPF/LL, GPRPESYREDI, and AAESGMDFSSRWFV, have also been marked (Figure 1). Phylogenetic analysis of Tres revealed that SlTre1 was grouped into a single subbranch and adjacent to SeTre1in *Spodoptera exigua*. SlTre2 was clustered within a subbranch together with SeTre2 in *S. exigua* (Figure 2).

### 3.2. Expression Patterns of SlTre1 and SlTre2

**Figure 3:**
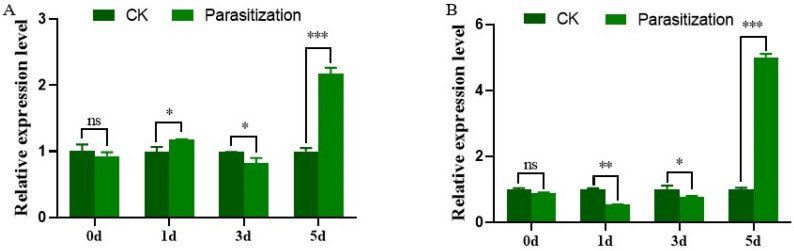
Expression levels of *SlTre1* (**A**) and *SlTre2* (**B**) in *S. litura* larvae after parasitization by the endoparasitoid *M. pulchricornis* at different time points. Significant differences in the expression levels of each target gene were compared using one-way analysis of variance (ANOVA). Significant differences are indicated by asterisks (* *p* < 0.01; ** *p* < 0.01; *** *p* < 0.001; ns: no significant differences, Table S4).

Parasitization had an effect on the expression of *SlTres* in *S. litura* larvae after they were parasitized by *M. pulchricornis* females. Both *SlTre1* and *SlTre2* were upregulated significantly five days after parasitization (Figure 3, Table S4). In addition, *SlTre1* was upregulated one day after parasitization but downregulated three days after parasitization (Figure 3A). The expression level of *SlTre2* was decreased one day and three days after parasitization (Figure 3B). At parasitism (0 day after parasitization), the expression of both *SlTre1* and *SlTre2* was not significantly different from the control groups (Figure 3).

### 3.3. Analysis of the Function of SlTres Using RNAi

**Figure 4:**
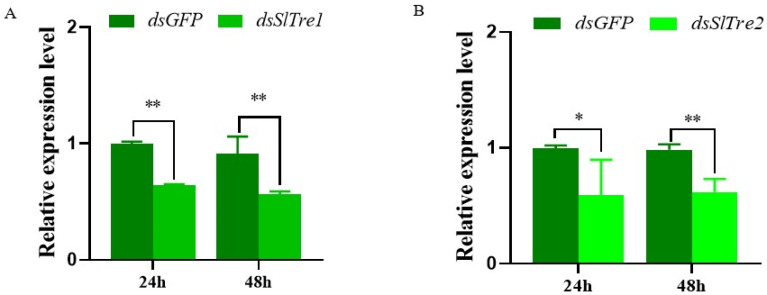
Expression levels of *SlTre1* (**A**) and *SlTre2* (**B**) after *dsSlTres* injection in parasitized third instar *S. litura* larvae. Significant differences in the expression levels of each target gene were compared using ANOVA. Significant differences are indicated by asterisks (* *p* < 0.05; ** *p* < 0.01, Table S4).

**Figure 5:**
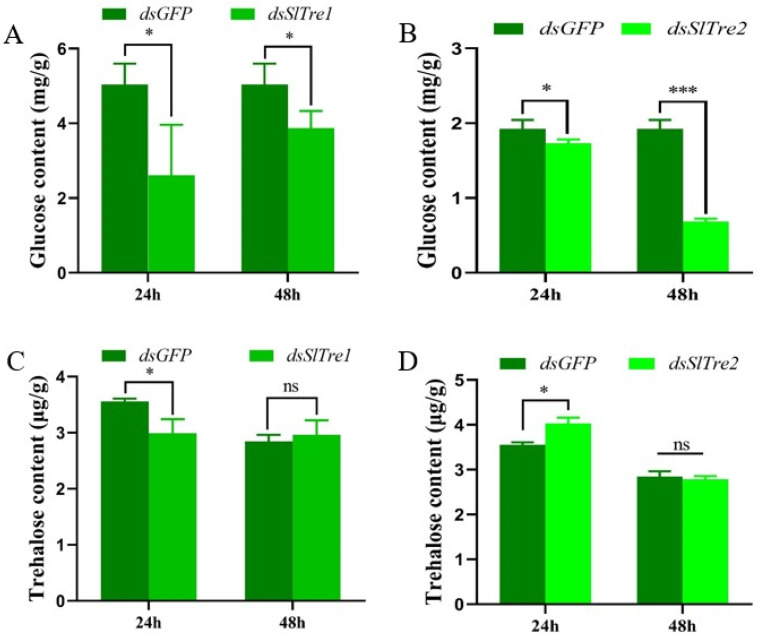
Glucose (**A**,**B**) and trehalose content (**C**,**D**) in parasitized *S. litura* larvae after RNAi at 24 and 48 h. Significant differences in the glucose and trehalose content were compared using ANOVA. Significant differences are indicated by asterisks (* *p* < 0.01; *** *p* < 0.001; ns: no significant differences, Table S4).

**Figure 6:**
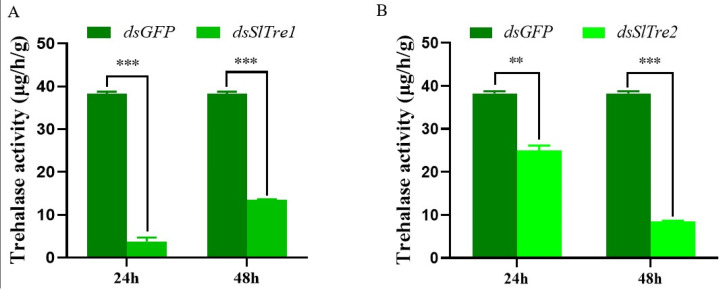
Trehalase activity in parasitized *S. litura* larvae after silencing *SlTre1* (**A**) and *SlTre2* (**B**) at 24 and 48 h. Significant differences in the trehalase activity were compared using ANOVA. Significant differences are indicated by asterisks (** *p* < 0.01; *** *p* < 0.001, Table S4).

Based on the results that *SlTre1* and *SlTre2* were upregulated significantly five days after parasitization obtained in Section 3.2, the subsequent functional validations were all conducted in the third instar *S. litura* larvae five days post-parasitization. Both *SlTre1* and *SlTre2* were successfully silenced in third instar parasitized *S. litura* larvae, with expression levels significantly decreased 24 and 48 h after dsRNA injection, compared to *dsGFP* injection groups (Figure 4A,B, Table S4). In order to test the effect of *SlTres* on trehalose metabolism in parasitized *S. litura* larvae, the content of trehalose and glucose in the bodies of parasitized *S. litura* larvae was determined. The results showed that 24 and 48 h after silencing *SlTre1* or *SlTre2*, the content of glucose, which is the hydrolysis product of trehalose, was significantly decreased compared to the control (Figure 5A,B, Table S4). Interestingly, the content of trehalose was only decreased significantly 24 h after silencing *Sltre1* (Figure 5C, Table S4); however, this reduction was not observed in *dsSlTre2*-injection groups (Figure 5D, Table S4) nor in the cohorts of 48 h after silencing *SlTre1* (Figure 5C). Furthermore, the activities of trehalase were notably reduced both in *SlTre1*- or *SlTre2*- silenced *S. litura* larvae, regardless of the elapsed duration after dsRNA injection (Figure 6A,B, Table S4).

**Figure 7:**
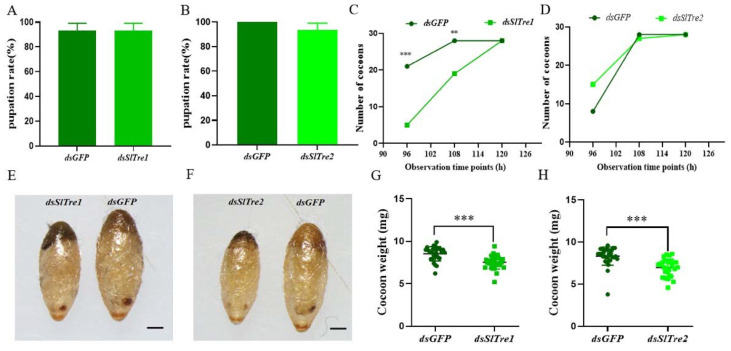
The pupation rate, duration from egg to cocoon, and morphology and weight of cocoons of parasitic wasp offspring egressed from parasitized *S. litura* larvae after they were knocking down *SlTre1* or *SlTre2*. Effect of silencing *SlTre1* or *SlTre2* on the pupation rate (**A**,**B**), durations from egg to cocoon (**C**,**D**), morphology of cocoons (**E**,**F**), and cocoon weight (**G**,**H**) in parasitized *S. litura* larvae. Significant differences were compared using ANOVA or chi-square test (for the data of duration from egg to cocoon). Significant differences are indicated by asterisks (** *p* < 0.01; *** *p* < 0.001; ns: no significant differences, Table S4); Scale bars in (**E**,**F**): 1 mm.

**Figure 8:**
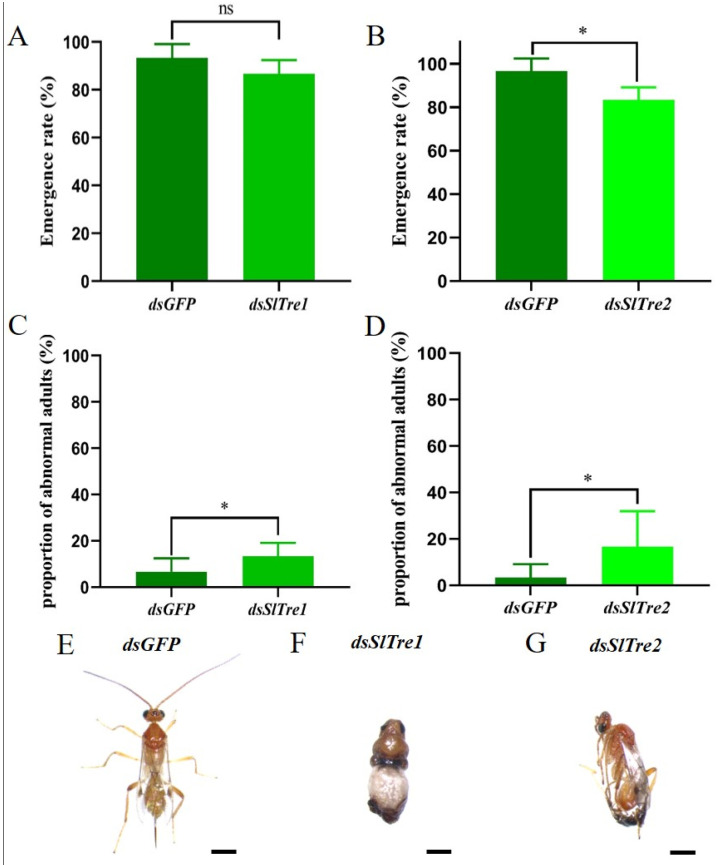
The emergence rate, proportion, and morphology of abnormal adults of parasitic wasp offspring egressed from parasitized *S. litura* larvae after silencing *SlTre1* or *SlTre2*. Effect of silencing *SlTre1* or *SlTre2* on emergence rate (**A**,**B**), the proportion of abnormal adults (**C**,**D**), and morphology of abnormal adults of parasitic wasp offspring egressed (**E**–**G**) from parasitized *S. litura* larvae. Significant differences were compared using ANOVA. Significant differences are indicated by asterisks (* *p* < 0.05; ns: no significant differences); Scale bars in (**E**–**G**): 2 mm.

**Figure 9:**
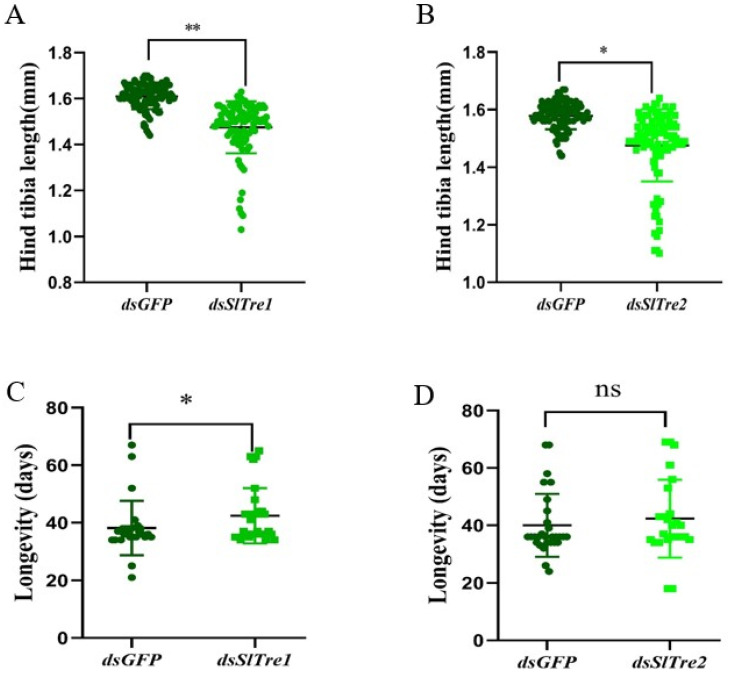
Effect of silencing *SlTre1* or *SlTre2* on the hind tibia length (**A**,**B**) and longevity (**C**,**D**) of parasitic wasp offspring egressed from parasitized *S. litura* larvae. Significant differences were compared using ANOVA. Significant differences are indicated by asterisks (* *p* < 0.05; ** *p* < 0.01; ns: no significant differences, Table S4).

In order to evaluate the effect of trehalase on parasitoid offsprings, the fitness parameters were examined. There was no significant difference in the pupation rate between *dsSlTre1* or *dsSlTre2* injection groups with control groups (Figure 7A,B, Table S4). Both *dsSlTre1* and *dsSlTre2* injection groups egressed a total of 28 offspring cocoons, and this number was equal with that in *dsGFP* injection groups. Despite this, as illustrated in Figure 7C, more cocoon formation was observed in control groups than in *dsSlTre1* injection groups at the observation time points of 96 h and 108 h, indicating that the offspring wasps took a longer time to reach the cocoon stage in the dsSlTre1 injection group than in the control. Therefore, the duration from eggs to cocoons was significantly prolonged in the *dsSlTre1* injection groups (Figure 7C); however, this delay was not observed in the dsSlTre2 injection groups (Figure 7D). In addition, the cocoons egressed from the *dsSlTre1* and *dsSlTre2* injection groups were smaller than those from the *dsGFP* injection groups (Figure 7E,F), and the weight of the cocoons egressed from the *dsSlTre1* and *dsSlTre2* injection groups were lighter than those from the *dsGFP* injection groups (Figure 7G,H, Table S4). When the immature parasitoids molted into the adult stage, there was no significant difference in the emergence rate between the *dsSlTre1* and *dsGFP* injection groups (Figure 8A, Table S4), but the emergence rate was lower in the *dsSlTre2* injection groups than that in the *dsGFP* injection groups (Figure 8B, Table S4). Meanwhile, more abnormal adults were observed both in the *SlTre1*- and *SlTre2*-silenced groups (Figure 8 C–G, Table S4). The adult body size, which was determined by the hind tibia length of offspring wasps egressed from silencing *SlTre1* and *SlTre2* host larvae, was remarkably smaller than in the control groups (Figure 9A,B, Table S4). Furthermore, the longevity of offspring adults was prolonged in the *SlTre1*-silenced groups (Figure 9C, Table S4); however, there was no significant difference in the longevity of offspring adults between the *dsSlTre2* and *dsGFP* injection groups (Figure 9D, Table S4).

## 4. Discussion

Trehalase is an important sugar metabolism enzyme in organisms, and it is the only reported hydrolase of trehalose in all organisms so far [2]. Since insect trehalase has two distinct forms, there are usually two genes encoding trehalase identified in insects [1,2]. In the present study, two trehalase genes, *SlTre1* and *SlTre2*, were identified from our previously constructed transcriptome dataset, and this number was consistent with the general rule. Multiple sequence alignment of trehalase amino acid sequences showed the presence of some of the general conserved signature motifs or structural domains, suggesting trehalase in *S. litura* may possess basic enzymatic properties. Phylogenetic analysis of trehalase amino acid sequences also showed the higher homology in noctuidae, in which trehalases were clustered into the same subbranches with trehalase in *S. exigua*.

Although the characterization and enzymatic properties of trehalase in *S. litura* has been reported in previous studies [19,20], the detailed function and molecular mechanism of trehalase in *S. litura* when they suffered from the infection of exogenous organisms, such as microbes or parasitoid wasps, has not been described yet. It is well-accepted that parasitoid wasps can induce great changes on the inner physiological states and alter the nutritional metabolism in their host insects [15]. When referring to this nutritional interaction between parasitoids and their hosts, the majority of previous studies focus on the variation in specific nutrition content. For instance, the content of triglycerides and glycogen in the host’s body fat is greatly decreased after parasitization by *Campoletis sonorensis*, but trehalose titers in hemolymph is increased [24]. Furthermore, this nutrition dynamic strictly depends on the developmental strategies of parasitoids [25]. Despite this, the molecular mechanism regulating this biochemical alternation still receives less attention.

With the aid of transcriptome sequencing technology, plenty of metabolism-related genes were identified from the host insect after they were parasitized by parasitic wasps.

For instance, a Kyoto Encyclopedia of Genes and Genomes (KEGG) analysis revealed that the sugar metabolism pathways were significantly enriched in the upregulated differentially expressed genes (DEGs) that were obtained by transcriptome sequencing in the midgut of *D. melanogaster* 24 h and 48 h post *L.* parasitization by *Leptopilina boulardi* [26]. Based on the transcriptomic analysis, Chen et al. [27] found that carbohydrate-metabolism-related Kyoto Encyclopedia of Genes and Genomes (KEGG) groups were enriched in *Ostrinia furnacalis* larvae parasitized by *Macrocentrus cingulum*, and trehalase genes were upregulated 48 h after parasitization. Similarly, in the present study, qRT-PCR validation revealed a typically significant increment in the expression levels of both *SlTre1* and *SlTre2* 5 d after parasitization, suggesting trehalase was significantly induced at this time point after parasitization. The reason of setting the fifth day after parasitization as the end time point of qRT-PCR validation was as follows. Generally, the female *M. pulchricornis* adults lay their eggs into the host bodies (at parasitism, 0d), and the eggs hatch 1~2d after parasitization. In most cases, the hatched larvae grow and develop until the eighth to tenth day after parasitization [28], and then they will egress from the host bodies and spin a cocoon to enter the pupal stage. However, in some scenarios, it is noticed that the development of offspring larvae is not quite uniform, and some could egress from the body and enter the pupal stage from the sixth or seventh day after parasitization [28]. Once the parasitoid larvae egressed from the host, the inner physiology of host larvae changed sharply, and they would be dead soon. Therefore, we selected the fifth day as the end time point. In addition, other studies demonstrated that trehalase can be induced in other insect species when confronted by stressors. For example, both high temperature and starvation can induce the expression of *BlTres* in bumblebee, *Bombus lantschouensis* [29]. By contrast, trehalase genes were upregulated after cold storage in *Harmonia axyridis* adults [30]. Zhao et al. [31] revealed that after various concentrations of trehalase inhibitor Validamycin A treatment, the expressions of *NlTRE1-1*, *NlTRE1-2*, and *NlTRE2* in rice brown planthopper *Nilaparvata lugens* were all upregulated, and they speculated that *TRE* genes attempt to synthesize more trehalase protein when the trehalase activities have been inhibited by Validamycin. Obviously, parasitization may be the key explanation for the upregualtion of *trehalase* expression pattern. Therefore, it is predicted that the expression of trehalase can be manipulated in response to both biotic and abiotic stressors.

Because of the uniqueness of trehalase in the hydrolysis of trehalose, we aimed to further explorer the exact role of trehalase in mediating the trehalose metabolism in *S. litura* larvae when they were parasitized by *M. pulchricornis* females. We successfully silenced both *SlTre1* and *SlTre2* by RNAi, and correspondingly, the trehalase activity was significantly decreased. Furthermore, the content of glucose, which is the hydrolysate of trehalose catalyzed by trehalase, was significantly decreased both in *dsSlTre1*- and *dsSlTre2*-injected host larvae. It should be noticed that glucose content levels in two dsGFP injection groups differed in Figure 5A,B. For this difference, there may be followed explanations. Firstly, the measurement of glucose content is determined as the content per capita (total glucose content divided by body weight of *S. litura* larva). It should be pointed out that the body weight of *S. litura* larvae varied significantly within the same instar (third instar used here), although their body size seems similar (similar body length or width). Thus, once calculating the content per capita, the result may differ greatly. Secondly, the difference between individuals and random error cannot be fully avoided in the process of glucose content measurement. In spite of this, the present results indicate the significant differences of glucose content between *dsTres* and *dsGFP* injection groups, suggesting that silencing SlTre genes significantly affected the glucose content in terms of content per capita. It can be speculated that although parasitization lays a strong modification on the host physiology including sugar metabolism [32,33,34], the trehalase still has consequences on the trehalose hydrolysis process. Interestingly, it is noticed that the content of trehalose was only decreased 24 h after knocking down *SlTre1*. Since trehalose metabolism is a bidirectional process consisting of synthesis and hydrolysis [23,35], it is reasonable that in the present parasitization association, after knocking down *SlTre2*, the trehalose-synthesis-related TPS or TPP [2] can be activated and more trehalose can be synthesized to compensate the deficit and ultimately meet the trehalose demand for the parasitoid offspring within the host larvae. Meanwhile, the trehalose compensation may also be attributed to the demand of host larvae. Further study is required to verify this feedback regulation hypothesis. Furthermore, the content of glucose and trehalose were determined at 24 h and 48 h after silencing, it is reasonably speculated that the sugar dynamics would be variable at other time points after dsRNA injection, although 24 and 48 h were successful silencing time points. Therefore, more measurement can be conducted to reveal further dynamics of glucose and trehalose content affecting by silencing *SlTres*.

Another important finding of the present study is that some key fitness traits of parasitoid offspring was strongly negatively affected after knocking down *SlTres* in host larvae. For instance, the emergence rates in *dsSlTre2*-injection, the proportion of abnormal adults, hind tibia length, and the cocoon weight in two *dsSlTres*-injection groups were all negatively affected. It is well established that host quality plays a strong impact on the fitness of the developing wasp offspring both in endo- and ecto-parasitoids [16,36]. For endoparasitoids, the wasp offspring usually develop for several days within the host, and different development stages require varied nutrition demands [37,38]. Particularly, after the immature parasitoids molt into the later stage during parasitization, they will consume more nutrition from host resources, and once the production or delivery of nutrition is impaired, the fitness of the wasp offspring will slow down the development [39]; even if they can successfully egress from the host and enter the next stage of their life cycle, more abnormal or smaller adults can be expected [40,41]. Therefore, the present results confirmed that trehalase is a vital regulatory nutrition factor in mediating trehalose metabolism in the association of parasitoids and their hosts and facilitating the development of the parasitoid offsprings.

## 5. Conclusions

In summary, the present study revealed that parasitization by *M. pulchricornis* can shape the expression pattern of two *trehalase* genes in *S. litura*. When trehalase was inhibited by silencing *SlTres*, the metabolism of trehalose in parasitized *S. litura* larvae would be affected and ultimately reduce the key fitness of parasitoid offspring. The results obtained here promote the understanding of the molecular mechanism in the nutritional interaction between parasitoid wasps with their host insects. More importantly, when referring the biological control practice acting by parasitoids, we can increase the expression of trehalase genes or change the dynamics of trehalose in host larvae to promote the breeding of parasitoid wasps or elevate the parasitization efficiency. In future studies, it is encouraged to reveal the details and further mechanisms of host trehalase regulation by parasitoids.

## Data Availability

All data presented in the work have been included in the article and can be provided by the authors upon reasonable request.

## Supplementary Materials

The following supporting information can be downloaded at: https://www.mdpi.com/article/10.3390/insects13090833/s1, Table S1: Species names and GenBank accession numbers of proteins from other species used for multiple alignment and phylogenetic tree analysis; Table S2: Primers used for real-time qRT-PCR of Trehalase genes in *Spodoptera litura*; Table S3: Oligonucleotide templates used to synthesize siRNA. Table S4: Statistical values of ANOVA in the present study.
