# Supplementary material for: Parasitoid Wasps Can Manipulate Host Trehalase to the Benefit of Their Offspring
Source: Insects. 2022 Sep 13;13(9):833. doi: 10.3390/insects13090833 (PMC9500599; doi:10.3390/insects13090833)
Supplement: Supplementary file 1 [file insects-13-00833-s001.zip › insects-1881357-supplementary.pdf]

Table S1. Species names and GenBank accessions of proteins from insect species used for multiple alignment and phylogenetic tree analysis.

Gene name	GenBank accessions
TcTre1	XP_015838405.1
TcTre2	XP_008200736.1
TcTre3	XP_015838404.1
TcTre4	XP_008197280.2
TcTre5	XP_973891.1
TcTre6	XP_008191350.2
BmTre	NP_001037458.1
AglTre	JQ246351
SeTre	EU427311
HaTre1	XP_021195450.1
HaTre2	XP_021194411.1
AaTre	XP_021705796.1
AmTre1	NP_001106141.1
AmTre2	XP_006560260.1
DmTre	NP_726024.1
LmTre	FJ795020.1
NlTre1	XP_022193105.2
NlTre2	XP_039290147.1
NlTre3	XP_039297459.1
OfTre1	EF426724.1
OfTre2	EF426723.1
CmTre1	KR149447.1
CmTre2	KR149447.1
GpTre1	MN915102.1
GpTre2	MN915101.1
SfTre1	XP_035432686.1
SfTre2	XP_035456757.1
SfTre3	XP_035432458.1
SfTre4	XP_035432458.1
SfTre5	XP_035433375.1
SfTre6	XP_035433201.1
AgTre	XP_320471.4

Table S2. Primers used for qRT-PCR of *Trehalase* genes in *Spodoptera litura.*

Gene name	Primer sequence（5'–3'）	Product size(bp)
Trehalase 1 F	ATCGTTATAGTTTGGTCCCT	135
Trehalase 1 R	TTCCTTTAGCGGTTTCTT	
Trehalase 2 F	CCAATCACAAGGACCCAG	113
Trehalase 2 R	CAGCCGCAGCTTTCAACT	
EF1 F	GAAACCGCTAAATACTATGTCA	161
EF1 R	CGGGTCTGTCCGTTCTTC	
GAPDH F	CTGCTAAAGCCGTAGGAAA	113
GAPDH R	CTGCTAAAGCCGTAGGAAA	

Table S3. Oligonucleotide templates used to synthesize siRNA.

Gene Name	Primer sequence（5'–3'）
Oligo- 1 Trehalase 1	GATCACTAATACGACTCACTATAGGGGGACACCACCCGATTTCAATT
Oligo-2 Trehalase 1	AATTGAAATCGGGTGGTGTCCCCCTATAGTGAGTCGTATTAGTGATC
Oligo-3 Trehalase 1	AAGGACACCACCCGATTTCAACCCTATAGTGAGTCGTATTAGTGATC
Oligo-4 Trehalase 1	GATCACTAATACGACTCACTATAGGGTTGAAATCGGGTGGTGTCCTT
Oligo- 1 Trehalase 2	GATCACTAATACGACTCACTATAGGGCCAGCGAGATCGCTACTAATT
Oligo- 2 Trehalase 2	AATTAGTAGCGATCTCGCTGGCCCTATAGTGAGTCGTATTAGTGATC
Oligo- 3 Trehalase 2	AACCAGCGAGATCGCTACTAACCCTATAGTGAGTCGTATTAGTGATC
Oligo- 4 Trehalase 2	GATCACTAATACGACTCACTATAGGGTTAGTAGCGATCTCGCTGGTT
Oligo-1GFP	GATCACTAATACGACTCACTATAGGGGGGATGTCTCACATCTTGTTT
Oligo-2GFP	AAACAAGATGTGAGACATCCCCCCTATAGTGAGTCGTATTAGTGATC
Oligo-3GFP	AAGGGATGTCTCACATCTTGTCCCTATAGTGAGTCGTATTAGTGATC
Oligo-4GFP	GATCACTAATACGACTCACTATAGGGACAAGATGTGAGACATCCCTT

Table S4. Statistical values of ANOVA in the present study.

		Treatment	F-value	df	p-value	95% C.I.	
						lower	upper
Relative expression level	SlTre1	0d	2.04	7	0.11	0.0688138	0.2936235
		1d	14.89	7	0.0182	-0.2797636	-0.0456531
		3d	12.14	7	0.0119	-0.3021293	-0.0534075
		5d	421.4	7	3.33E-05	1.021293	1.340746
	SlTre2	0d	0.16	7	0.21	-0.5216307	-0.4145546
		1d	91.19	7	0.00672	0.1262028	0.2296699
		3d	12.78	7	0.0233	-0.4225094	-0.0531486
		5d	402.3	7	3.70E-07	3.829535	4.180167
	SlTre1	24h	760.1	7	1.03E-05	-0.3590172	-0.3228618
		48h	15.59	7	0.0168	-0.591325	-0.1030604
	SlTre2	24h	5.087	7	0.00871	-0.9055588	0.9374615
		48h	30.63	7	0.00521	-0.5638709	-0.1871251
Glucose content	SlTre1	24h	6.695	7	0.0609	-0.5458466	0.01923859
		48h	7.906	7	4.82E-02	-2.325425	-0.0146874
	SlTre2	24h	7.578	7	0.0512	-0.4099499	0.0017479
		48h	287	7	7.12E-05	-1.438494	-1.033362
Trehalose content	SlTre1	24h	9.326	7	3.79E-02	0.06202438	1.30374
		48h	0.156	7	0.713	-0.8726575	0.6551388
	SlTre2	24h	5.386	7	0.0811	-0.160258	1.792689
		48h	0.144	7	7.23E-01	-0.294401	0.3876957
Trehalase activity	SlTre1	24h	3454	7	5.02E-07	-35.71618	-32.49394
		48h	321.2	7	5.70E-05	-15.21831	-11.13547
	SlTre2	24h	222.9	7	6.52E-04	-15.6124	-10.12573
		48h	3589	7	4.65E-07	-30.86082	-28.12715
Pupation rate		SlTre1	0	28	1.00E+00	-13.08834	13.08834
		SlTre2	4	28	1.16E-01	-15.92152	2.58819
cocoon weight		SlTre1	20.75	28	3.03E-05	-1.440179	-0.559821
		SlTre2	24.39	28	7.45E-06	-1.923979	-0.8136405
Emergence rate		SlTre1	2	28	2.30E-01	-19.75501	6.421678
		SlTre2	8	28	4.74E-02	-26.42168	-0.2449888
Proportion of abnormal adults		SlTre1	2	28	2.30E-01	-6.421678	19.75501
		SlTre2	2	28	2.30E-01	-12.84336	39.51002
Hind tibia length		SlTre1	116.1	28	<2e-16	-0.159473	-0.1101348
		SlTre2	57.26	28	1.41E-12	-0.127004	-0.0744926
Longevity		SlTre1	2.728	28	1.05E-01	-0.9128567	9.424793
		SlTre2	0.5	28	4.83E-01	-4.340263	9.060263
